# Primed CRISPR-Cas Adaptation and Impaired Phage Adsorption in Streptococcus mutans

**DOI:** 10.1128/mSphere.00185-21

**Published:** 2021-05-19

**Authors:** Cas Mosterd, Sylvain Moineau

**Affiliations:** a Département de Biochimie, de Microbiologie, et de Bio-informatique, Faculté des Sciences et de Génie, Université Laval, Québec City, Québec, Canada; b Groupe de Recherche en Écologie Buccale, Faculté de Médecine Dentaire, Université Laval, Québec City, Québec, Canada; c Félix d’Hérelle Reference Center for Bacterial Viruses, Université Laval, Québec City, Québec, Canada; Escola Paulista de Medicina/Universidade Federal de São Paulo

**Keywords:** phage, CRISPR-Cas, *Streptococcus mutans*, priming, adaptation, adsorption, resistance, bacteriophages

## Abstract

Streptococcus mutans strain P42S possesses a type II-A CRISPR-Cas system that protects against phage infection and plasmid transformation. The analysis of 293 bacteriophage-insensitive mutants (BIMs) obtained upon exposure to the virulent phage M102AD revealed the acquisition of 399 unique spacers, including several ectopic spacer acquisitions and a few cases of native spacer deletions. The acquisition of multiple spacers was also observed and appears to be mostly due to priming, which has been rarely reported for type II-A systems. Analyses of the acquired spacers indicated that 88% of them are identical to a region of the phage M102AD genome. The remaining 12% of spacers had mismatches with the phage genome, primarily at the 5′ end of the spacer, leaving the seed sequence at the 3′ end largely intact. When a high multiplicity of infection (MOI) was used in the phage challenge assays, we also observed the emergence of CRISPR BIMs that, in addition to the acquisition of new spacers, displayed a reduced phage adsorption phenotype. While CRISPR-Cas and adsorption resistance work in tandem to protect S. mutans P42S against phage M102AD, nonidentified antiviral mechanisms are also likely at play in this strain.

**IMPORTANCE** Bacteria are under the constant threat of viral predation and have therefore developed several defense mechanisms, including CRISPR-Cas systems. While studies on the mode of action of CRISPR-Cas systems have already provided great insights into phage-bacterium interactions, still more information is needed on the biology of these systems. The additional characterization of the type II-A CRISPR-Cas system of Streptococcus mutans P42S in this study provides novel information on the spacer acquisition step, especially regarding protospacer-adjacent motif (PAM) recognition, multiple-spacer acquisition, and priming.

## INTRODUCTION

Similar to all life forms, bacteria are under the constant threat of viral infection. To prevent infection by bacterial viruses, called bacteriophages or phages, bacteria encode multiple antiviral defense mechanisms. These antiviral defense mechanisms can target any of the multiple steps of the phage replication cycle. As a first line of defense, phage adsorption to the cell surface can be prevented through several means (1). If the infection is not halted at this stage and the phage manages to inject its genome, a wide variety of antiviral defense mechanisms, including CRISPR-Cas systems, may interfere with the replication of the phage genome (2, 3). CRISPR is an acronym for clustered regularly interspaced short palindromic repeats. Along with CRISPR-associated (Cas) proteins, they help to protect bacteria from invasion by nucleic acids such as phage genomes (4). The gene composition of these CRISPR-Cas systems is highly diverse and led to their current classification into two classes, six types, and several subtypes (5). Overall, they have been detected in approximately 40 to 45% of all bacterial species (5), including Streptococcus mutans (6).

S. mutans is an important member of the oral microbiota as it is a colonizer of the tooth surface and also the main cause of dental caries. Although not life-threatening, this disease is a health concern as well as a financial burden (7). Bacterial viruses are also members of the oral ecosystem. Yet despite various attempts, very few virulent phages infecting S. mutans have been isolated and reported in the literature (8, 9). A complicating factor is that S. mutans phages appear to have a very narrow host range (8, 10). However, sequencing of human salivary DNA predicted a high number of streptococcal phages in the oral cavity but mostly as prophages (11).

CRISPR-Cas systems naturally function through a multistep process. The first step, called the adaptation phase, is the least understood. Two genes, called *cas1* and *cas2*, are known to be essential for adaptation and are found in almost all types of CRISPR-Cas systems (5, 12). In the II-A subtype systems, such as those found in S. mutans strains, two additional genes are needed for adaptation, namely, *csn2* and *cas9* (13). CRISPR loci typically contain spacers between repeats that match mobile genetic elements such as phage genomes, indicating past phage-bacterium interactions and novel spacer acquisition or immunities. However, new spacer acquisition events following phage infection are seldom observed under laboratory conditions. Nevertheless, CRISPR-Cas systems have been found to acquire novel spacers in some experimental settings. For example, spacer acquisition following phage challenge has been observed for type II-A systems (4, 6, 14) and from plasmids in type II-C systems (15). Under specific conditions, spacer acquisition has also been observed in type I-B (16), I-E (17, 18), and I-F (19) systems.

Spacer acquisition can either be naive (from a phage genome that has never been encountered before) or primed. This primed adaptation refers to the acquisition of new spacers from a matching nucleic acid source that was previously the subject of a spacer acquisition event in the same cell. The acquisition of multiple spacers has the advantage of increasing the overall phage resistance of a bacterial cell (4, 20). In addition, priming is a phenomenon that increases the frequency of spacer acquisition under laboratory conditions (21). Although primarily observed in type I systems (16–19), priming has recently been associated with type II-A systems by bioinformatic analyses (22) as well as, to a certain degree, experimentally with CRISPR-Cas systems found in Streptococcus pyogenes (23) and Streptococcus thermophilus (24).

The spacer acquisition activity of type II-A CRISPR-Cas systems in S. mutans has already been demonstrated from phage genomes (6, 14) and plasmids (25), including the acquisition of multiple spacers in some cases (14). Here, we further investigated spacer acquisition in the type II-A CRISPR-Cas system of S. mutans, including to see if priming is occurring and influencing the overall antiviral response of the cells.

## RESULTS

### Spacer sizes and PAM preferences of the type II-A CRISPR-Cas system of S. mutans P42S.

In this study, we generated 174 new bacteriophage-insensitive mutants (BIMs) that have acquired spacers following infection of the wild-type (WT) phage-sensitive strain S. mutans P42S with the virulent phage M102AD under various conditions. The addition of these BIMs to the 119 BIMs obtained in a previous study (14) resulted in a set of 293 BIMs that were further analyzed. Altogether, these BIMs acquired 399 unique spacers. The spacer size ranged from 28 to 32 bp, with the majority (279/399; 70%) of them being 30 bp in length. Also, 105 spacers were 31 bp (26%), 11 were 32 bp (3%), 3 were 29 bp (1%), and 1 was 28 bp (<1%) in length.

Of the 399 acquired spacers, 352 (88%) were identical to short genomic regions of phage M102AD, and 38 (9%) had one to three mismatches, with 31 of them having only one mismatch. One spacer had 9 mismatches with a region of the phage M102AD genome. Two spacers (1%) perfectly matched the genome of S. mutans P42S. Surprisingly, the remaining six spacers (2%) did not match any known sequence. Overall, we could retrieve the origin of 393/399 (98%) of the acquired spacers.

**FIG 1:**
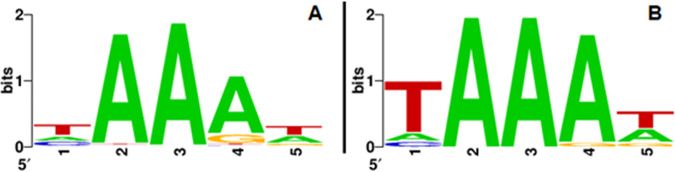
PAMs flanking all protospacers (A) and flanking the protospacers matching spacers acquired only during single-spacer acquisition events (B).

We identified a protospacer-adjacent motif (PAM) sequence flanking the 393 protospacers. Among these protospacers, 95% (375/393) were flanked by the 5′-NAA-3′ motif, while 78% (306/393) were flanked by the nucleotide sequence 5′-NAAA-3′ (Fig. 1). While the most commonly observed PAM was previously identified as 5′-TAAAT-3′ (14), here, the most frequently observed PAM was 5′-TAAAA-3′ (17%; 66/393) (Table 1). The motif 5′-TAAAT-3′ was associated with 13% of the protospacers (52/393). When considering only the BIMs that have acquired a single spacer, 100% (68/68) of the protospacers were flanked by 5′-NAA-3′. The PAMs 5′-TAAAA-3′ and 5′-TAAAT-3′ were both observed in 32% (22/68) of the cases. An overview of all the PAMs is shown in Table 1.

**TABLE 1:** PAMs flanking the targeted protospacers in the phage M102AD genome

No. of acquired spacers	PAM (5′–3′) flanking the targeted protospacer	PAM frequency in the genome of phage M102AD	Ratio of no. of acquired spacers/total no. of acquired spacers (393)	Ratio of no. of spacers acquired/no. of PAMs in phage M102AD genome
66	TAAAA	212	0.168	0.31
52	TAAAT	81	0.132	0.65
40	AAAAT	263	0.102	0.15
33	CAAAT	123	0.084	0.27
29	TAAAG	136	0.074	0.21
26	CAAAA	249	0.066	0.10
20	AAAAA	336	0.051	0.06
20	TAAGT	68	0.051	0.29
9	CAAAG	157	0.023	0.06
7	AAAGT	141	0.018	0.05
7	CAAGT	77	0.018	0.09
6	AAAAG	202	0.015	0.03
6	GAAAA	184	0.015	0.03
6	GAAAT	125	0.015	0.05
4	AAAAC	180	0.010	0.02
4	TAAGG	25	0.010	0.16
4	TTAAA	216	0.010	0.02
4	CAAAC	107	0.010	0.04
3	TAAAC	92	0.008	0.03
3	TAACT	87	0.008	0.03
3	TGAAA	128	0.008	0.02
2	AAATT	183	0.005	0.01
2	AAATC	175	0.005	0.01
2	AAAGA	196	0.005	0.01
2	AAAGG	73	0.005	0.03
2	TAATT	136	0.005	0.02
2	TAACA	88	0.005	0.02
2	TAAGA	78	0.005	0.03
2	TAAGC	103	0.005	0.02
2	CAAGA	112	0.005	0.02
2	CAAGG	45	0.005	0.04
2	GAAAC	86	0.005	0.02
1	AAATA	121	0.003	0.01
1	AAATG	112	0.003	0.01
1	AAACC	64	0.003	0.02
1	AAGTG	64	0.003	0.02
1	AAGCT	176	0.003	0.01
1	ATAAA	111	0.003	0.01
1	TAATA	96	0.003	0.01
1	TAATG	71	0.003	0.01
1	TATAT	101	0.003	0.01
1	TACAG	74	0.003	0.01
1	TCAAA	212	0.003	<0.01
1	TCGCC	33	0.003	0.03
1	TGAAG	86	0.003	0.01
1	CAATT	106	0.003	0.01
1	CAACG	35	0.003	0.03
1	CTAAA	122	0.003	0.01
1	CTATT	68	0.003	0.02
1	CCAAG	44	0.003	0.02
1	GAAGT	70	0.003	0.01

**FIG 2:**
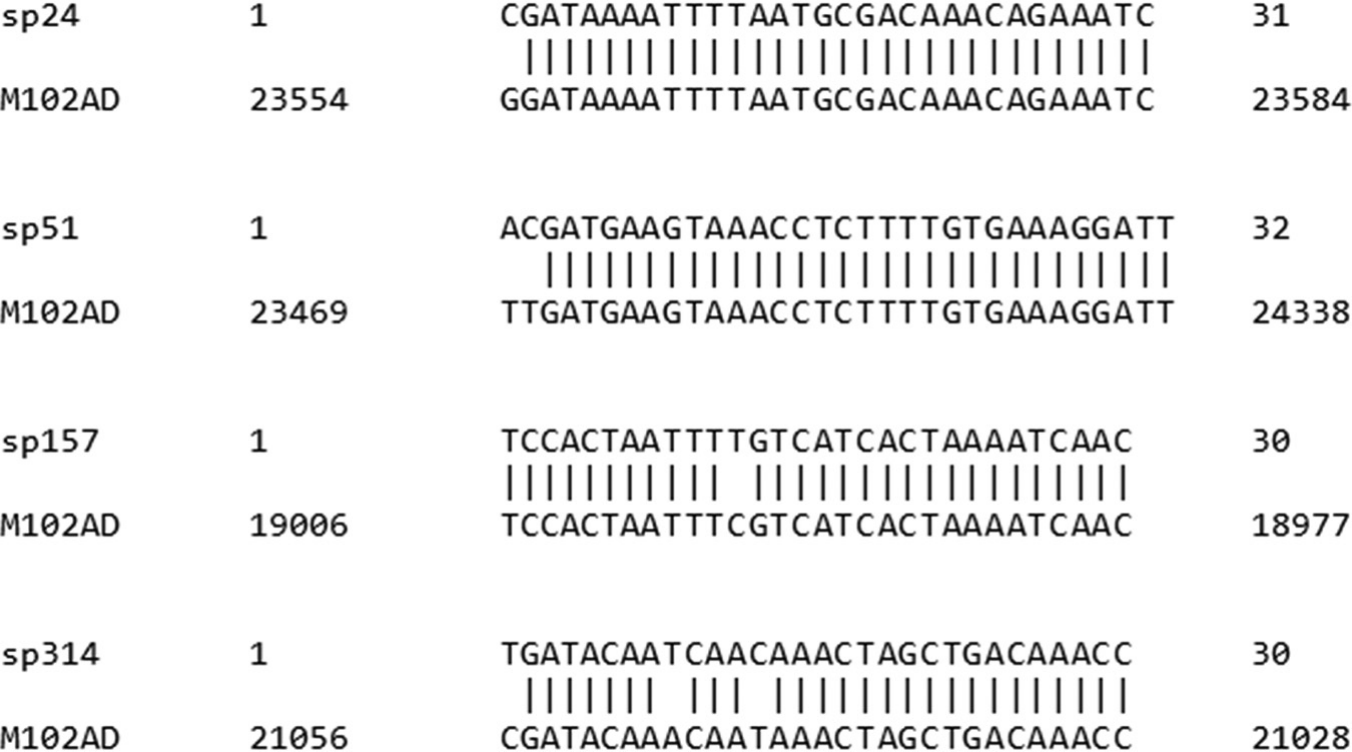
Sequences of non-perfectly matching spacers in BIMs of S. mutans and their targets on the genome of phage M102AD.

Among those spacers with one to three mismatches, 34 out of the 38 (92%) had a mismatch at the first base pair of the spacer. Interestingly, 27/31 spacers that had a 1-bp mismatch were in fact 31 bp long (and 1/31 was 32 bp long) and contained a stretch of 30 bp perfectly matching a region of the phage genome. Similarly, all five spacers with two mismatches were 32 bp long and included a 30-bp stretch identical to the phage genome. Therefore, 384/399 of the acquired spacers had a perfect match of at least 30 bp to the phage genome (Fig. 2).

Of note, all 27 spacers of 31 bp in length that possessed a mismatch at the first base pair had a C at that position. In the 4 spacers of 32 bp in length with two mismatches at the first 2 bp, these first 2 bp were always AC.

### Ectopic spacer acquisition.

The CRISPR array of the wild-type phage-sensitive S. mutans strain P42S contains five native spacers and six repeats. Usually, new spacers are added at the 5′ end of the CRISPR array in bacterial cells surviving phage infection. However, the acquisition of spacers within the CRISPR array (ectopic) has been observed with the type II-A systems of S. pyogenes (26), S. thermophilus (27–29), and S. mutans (14). Among our set of BIMs, 30% (89/293) have acquired ectopic spacers. Typically, only one ectopic spacer was acquired in each of the BIMs, but multiple ectopic spacers were acquired in 12% (11/89) of them. In the vast majority of the BIMs that have acquired ectopic spacers (81/89; 91%), at least one additional spacer was also integrated at the 5′ end of the array. All ectopic spacers, except one, were acquired between the native spacers 4 and 5. The other was acquired between spacers 1 and 2.

A total of 78 different spacers were acquired ectopically. Of these, 25 (25/78; 32%) have also been acquired at the 5′ end of the CRISPR locus in other BIMs. Of the remaining 53 ectopically acquired spacers, 52 have an identifiable PAM associated with the corresponding protospacer. PAM preferences did not differ significantly compared to the PAMs flanking the protospacers targeted by the spacers acquired at the 5′ end of the array, as 91% (47/52) were flanked by 5′-NAA-3′, and 67% (35/52) were flanked by 5′-NAAA-3′.

**FIG 3:**
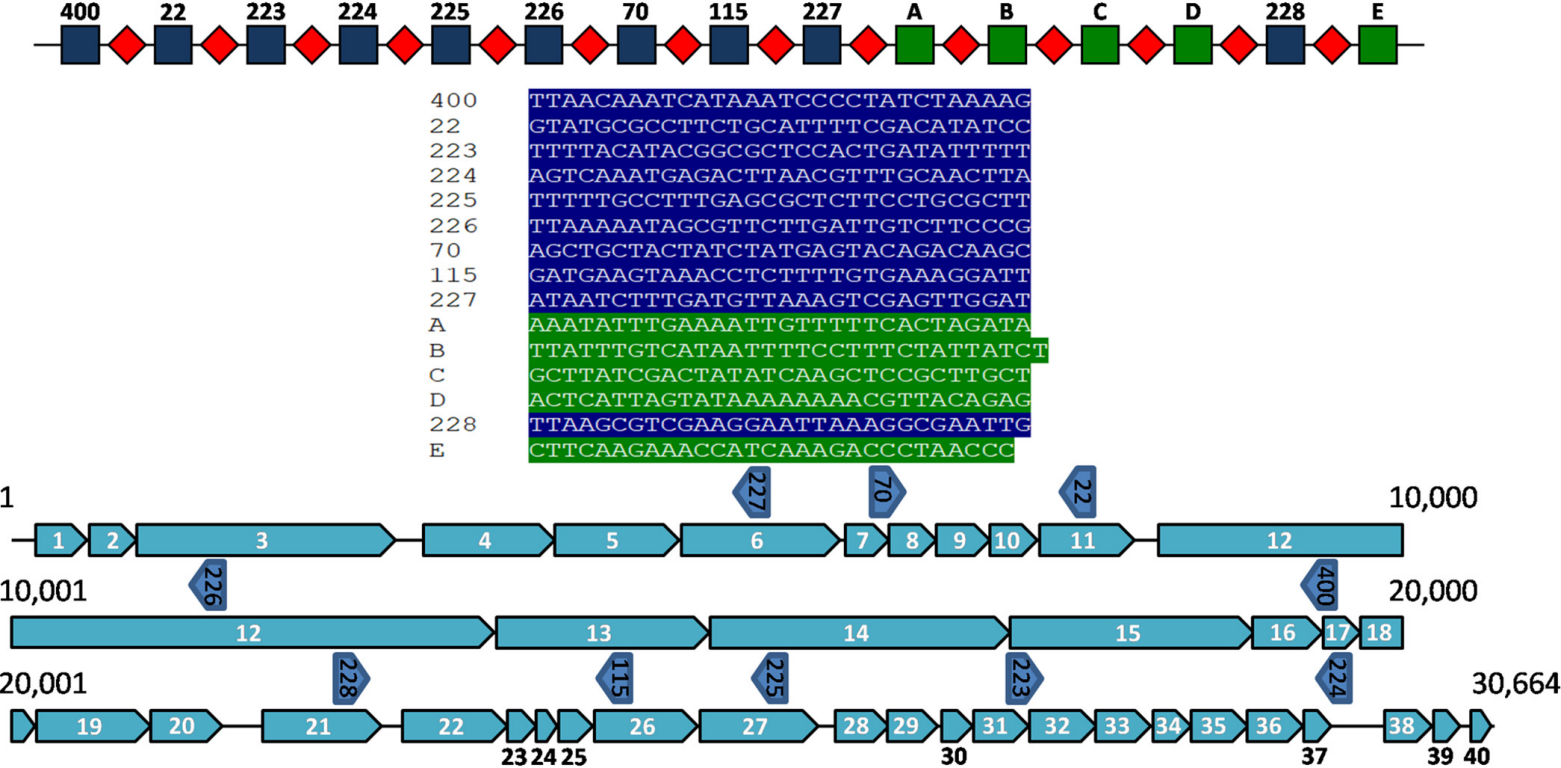
CRISPR locus of S. mutans BIM 2.2-43CA and the protospacers on the genome of phage M102AD. At the top, repeats are illustrated as red diamonds, and the native spacers in the wild-type CRISPR locus of S. mutans P42S are shown as green squares. Newly acquired spacers are displayed as dark blue squares. The sequences of the spacers are listed below the CRISPR locus. The color code corresponds to the one used in the CRISPR locus. The genome of phage M102AD is at the bottom. The protospacers are highlighted by short arrows above the phage genes and are identified by the corresponding spacer numbers. The 10 acquired spacers targeted phage genes coding for hypothetical proteins (open reading frame 17 [ORF17], ORF27, and ORF31), a major tail protein (ORF11), a tape measure protein (ORF12), a DNA packaging protein (ORF7), a single-strand annealing protein (ORF26), a capsid protein (ORF6), and a replisome organizer (ORF21) as well as a noncoding region (between *orf37* and *orf38*).

Among the BIMs isolated during this study, one BIM acquired 10 new spacers during a single infection assay with phage M102AD. Nine new spacers were acquired at the 5′ end of the CRISPR array, and one was acquired ectopically. The CRISPR locus of this BIM and the targets of the acquired spacers are highlighted in Fig. 3.

Finally, we also observed the deletion of four of the five native spacers in two BIMs (BIM 4.6-9B and BIM 9.2-8C). Both BIMs acquired one new spacer targeting the phage M102AD genome while maintaining only the WT spacer at the 3′ end of the locus.

### Acquisition of non-perfectly matching spacers.

Of the 293 characterized BIMs, 58 acquired at least one spacer that did not perfectly match a region of the phage genome. The vast majority of these BIMs (51/58) also acquired at least one other spacer perfectly matching a region of the phage M102AD genome. Among those 51 BIMs, 19 (37%) acquired the non-perfectly matching spacers at the 5′ end of the CRISPR locus, while 12 of them acquired multiple spacers at the 5′ end, and the non-perfectly matching spacers were located furthest away from the 5′ end of the locus. Additionally, 11 BIMs acquired non-perfectly matching spacers ectopically.

**FIG 4:**
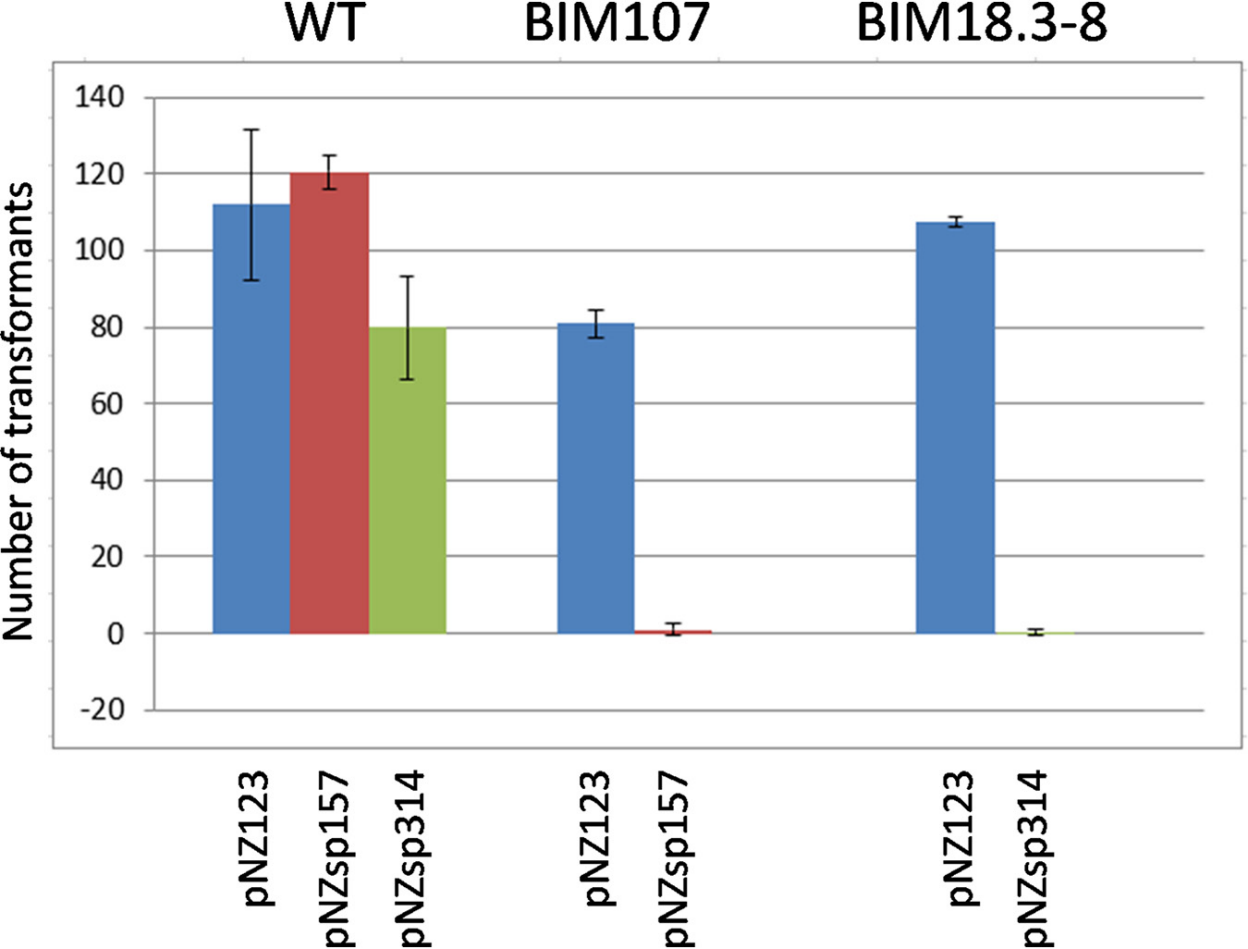
Interference efficacy of non-perfectly matching spacers. Plasmids containing the targeted protospacers were transformed into wild-type S. mutans P42S or into BIMs containing the nonmatching spacer. The numbers of transformants are per microgram of plasmid DNA used in the transformation assay. pNZ123 carries no protospacer and was used as a control. pNZsp157 carries the protospacer non-perfectly matching spacer sp157. pNZsp314 carries the protospacer non-perfectly matching spacer sp314.

Only 7 BIMs acquired a single, non-perfectly matching spacer, including 5 that acquired a spacer with one, two, or three mismatches, while 2 acquired a spacer with low sequence identity to the phage M102AD genome. The interference activity of two non-perfectly matching spacers, sp157 (one mismatch) and sp314 (three mismatches), was examined using a plasmid transformation assay. The mismatches are highlighted in Fig. 2. In these assays, plasmid constructs carrying a protospacer derived from phage M102AD were transformed into BIMs carrying a targeting but non-perfectly matching spacer in their CRISPR array. The empty vector pNZ123, which does not contain any protospacer, was also transformed into these BIMs. pNZ123, pNZsp157, and pNZsp314 were all transformable into the wild-type strain S. mutans P42S (Fig. 4). However, the transformation of pNZsp157 into BIM 107, which had previously acquired spacer 157, was negligible. The efficiency of the transformation of pNZsp314 into BIM 18.3-8 was equally close to zero. pNZ123 could be transformed efficiently into both BIMs. These data indicate that these non-perfectly matching spacers are still providing CRISPR-based interference (Fig. 4).

### Priming.

BIMs were also examined to determine whether priming could have taken place in the type II-A system of S. mutans P42S. Of the 293 BIMs, 193 of them acquired multiple spacers. In the case of ectopic spacers, it could not be determined which spacer was acquired first. As such, we subtracted 81 BIMs that also acquired ectopic spacers as well as 1 BIM in which the second spacer was not targeting the genome of phage M102AD and could therefore not be used for the priming analysis. Of the remaining 111 BIMs, 59 BIMs have acquired two spacers targeting the phage M102AD genome and are located at the 5′ end of the CRISPR locus, and 52 BIMs acquired more than two spacers. We assumed that the spacer closest to the native spacers was acquired first.

**FIG 5:**
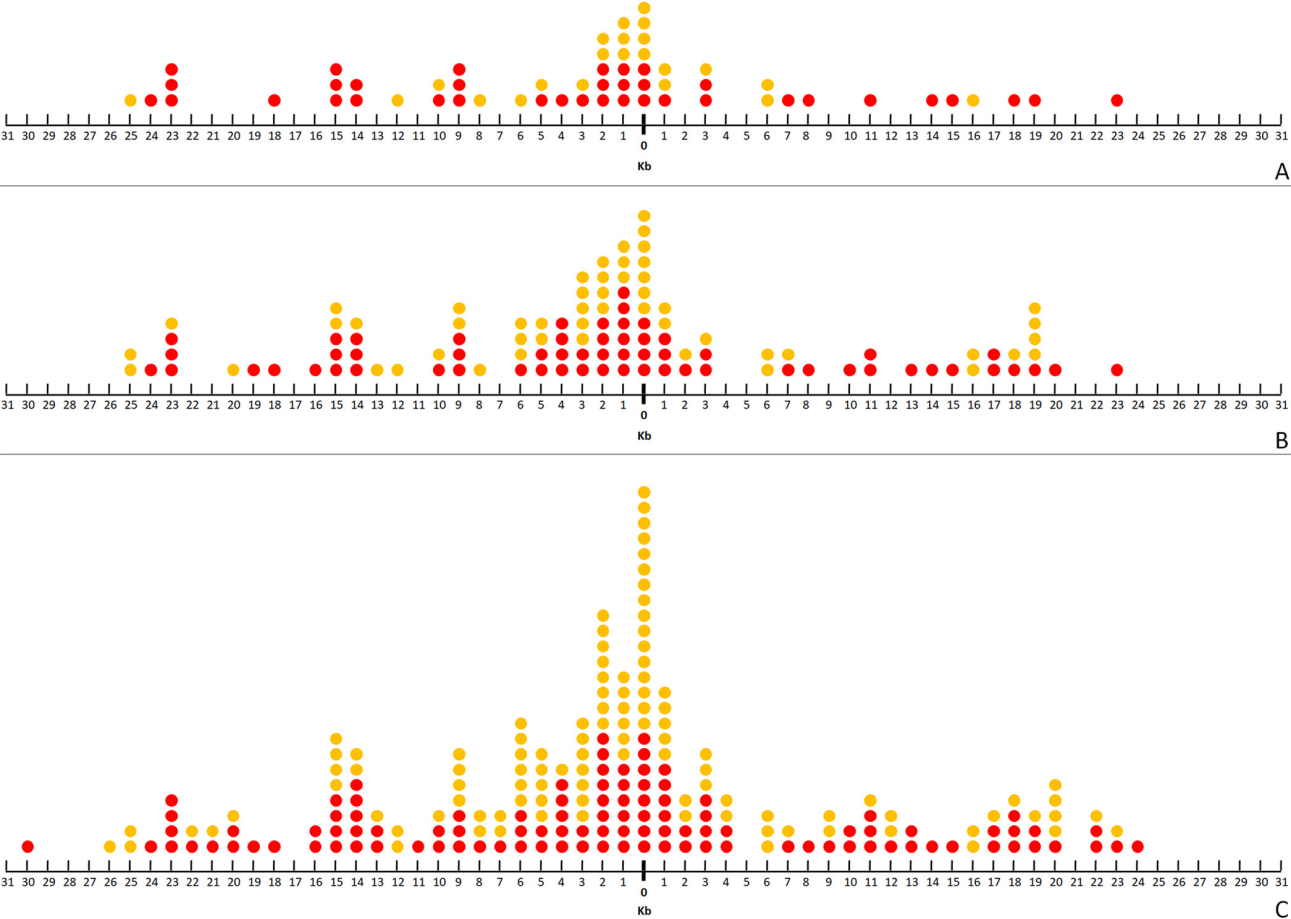
Priming in Streptococcus mutans P42S. Second spacers acquired from the same strand as the first spacer are illustrated as red dots, and those acquired from the other strand are illustrated as yellow dots. (A) The 59 second spacers (the one at the 5′ end of the CRISPR array) acquired by BIMs with only two new spacers at the 5′ end of the array. (B) The 111 second spacers of the BIMs that have acquired multiple spacers. (C) The 211 spacers acquired by BIMs that have acquired multiple spacers at the 5′ end of their loci. The *x* axis represents the distance in the phage M102AD genome between the protospacers matching the second and first spacers acquired by the BIMs. On the right of value 0, the protospacers matching the second acquired spacers are found downstream of the protospacer matching the first acquired spacer. On the left of value 0, those protospacers are found upstream of the protospacer matching the first acquired spacer. Those at value 0 are found <500 bp upstream or downstream from the protospacer matching the first acquired spacer. Those at value 1 are found ≥500 bp and <1,500 bp downstream of the protospacer matching the first acquired spacer.

First, we focused on the 59 BIMs that have acquired two spacers. Specifically, we looked at the distance on the phage M102AD genome between the protospacers targeted by the first and second acquired spacers (Fig. 5A). In 36% of the cases (21/59), the second spacer (the one at the 5′ end of the CRISPR array) was acquired from a protospacer that was within 2.5 kb from the protospacer matching the first (original) spacer. In 12% of the cases (7/59), the distance was less than 500 bp. Of the 59 spacers, 37 (63%) were acquired from the same strand as the original spacer, and 22 (37%) spacers originated from the other strand. Of the 37 spacers acquired from the same strand, 10 (27%) were acquired from within 2.5 kb from the original spacer, while 11 (50%) of the 22 spacers from the other strand were acquired from within 2.5 kb.

Next, we performed the same analyses with the 52 BIMs that had acquired more than two spacers. In this case, only the second spacer next to the last native spacer was considered in the analysis since this one was presumably acquired after the first new spacer. In addition to the 59 spacers described above, these 52 additional spacers gave us a set of 111 spacers to analyze for possible priming (Fig. 5B). The percentages remained essentially the same with this set of spacers. In 32% of the cases (35/111), the protospacer matching the second acquired spacer was within 2.5 kb from the protospacer matching the first spacer, and in 10% of the cases (11/111), the protospacer was within 500 bp. Of the 111 spacers, 60 (54%) of them were acquired from the same strand as the original spacer, and 51 (46%) spacers originated from the other strand. These percentages are closer to each other than the set of 59 spacers described above. Spacers acquired from the same strand were acquired from within 2.5 kb in 30% of the cases (18/60) and from within 500 bp in 7% (4/60). As for the other strand, 33% of the spacers (17/51) were acquired from within 2.5 kb, and 14% (7/51) were acquired from within 500 bp.

Finally, when considering all the spacers acquired by these 111 BIMs, 32% of the spacers (67/211) were found within 2.5 kb from the original spacer, and 11% (24/211) were found within 500 bp. A total of 30 spacers out of 106 (28%) were acquired from the same strand (30/106) and were within 2.5 kb from the original spacer, and 8% (8/106) were within 500 bp. Of the spacers acquired from the other strand, 35% (37/105) were acquired within 2.5 kb, and 15% (16/105) were acquired within 500 bp (Fig. 5C).

**FIG 6:**
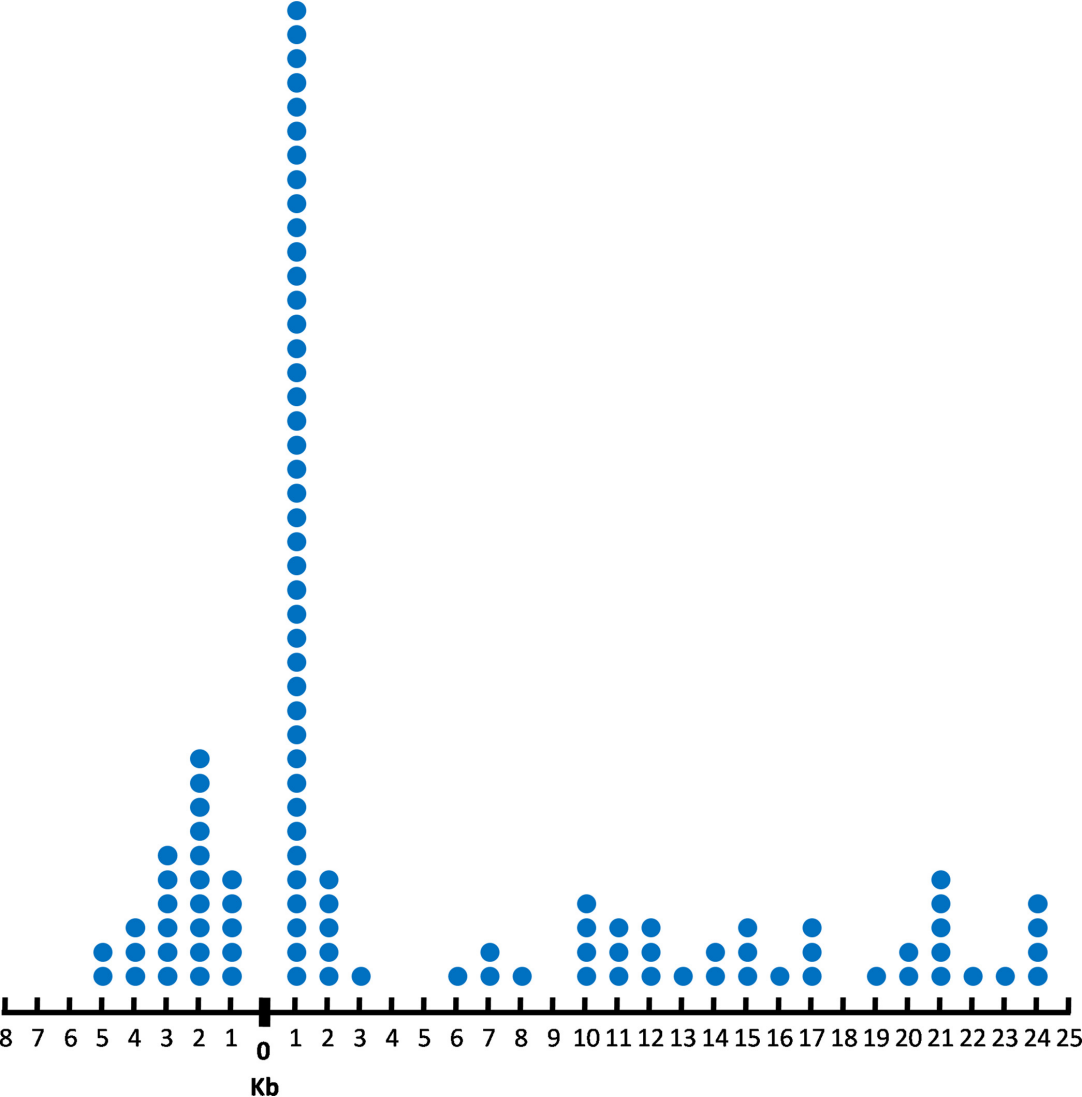
Distance in the phage M102AD genome between the protospacers matching the first spacers acquired by the BIMs and the protospacer partially matching native spacer 3 in the CRISPR array of S. mutans P42S. The approximate distance is in kilobases and is illustrated on the *x* axis with respect to value 0, which is the position of the protospacer matching spacer 3. On the right of value 0, the protospacers are found downstream of the protospacer matching spacer 3, and on the left of value 0, those spacers are found upstream of the protospacer matching spacer 3.

We also noticed that spacer 3 in the native CRISPR array of S. mutans P42S partially matched a region of the phage M102AD genome (bp 25002 to 24973). This spacer has mismatches with the phage M102AD genome at positions 2, 8, 23, and 27. This raised the question of whether this partial identity could have played a role in priming. Our analysis revealed that 55% of the protospacers matching the acquired spacers (61/111) were within 2.5 kb of the region on the phage M102AD genome matching spacer 3 (Fig. 6).

**FIG 7:**
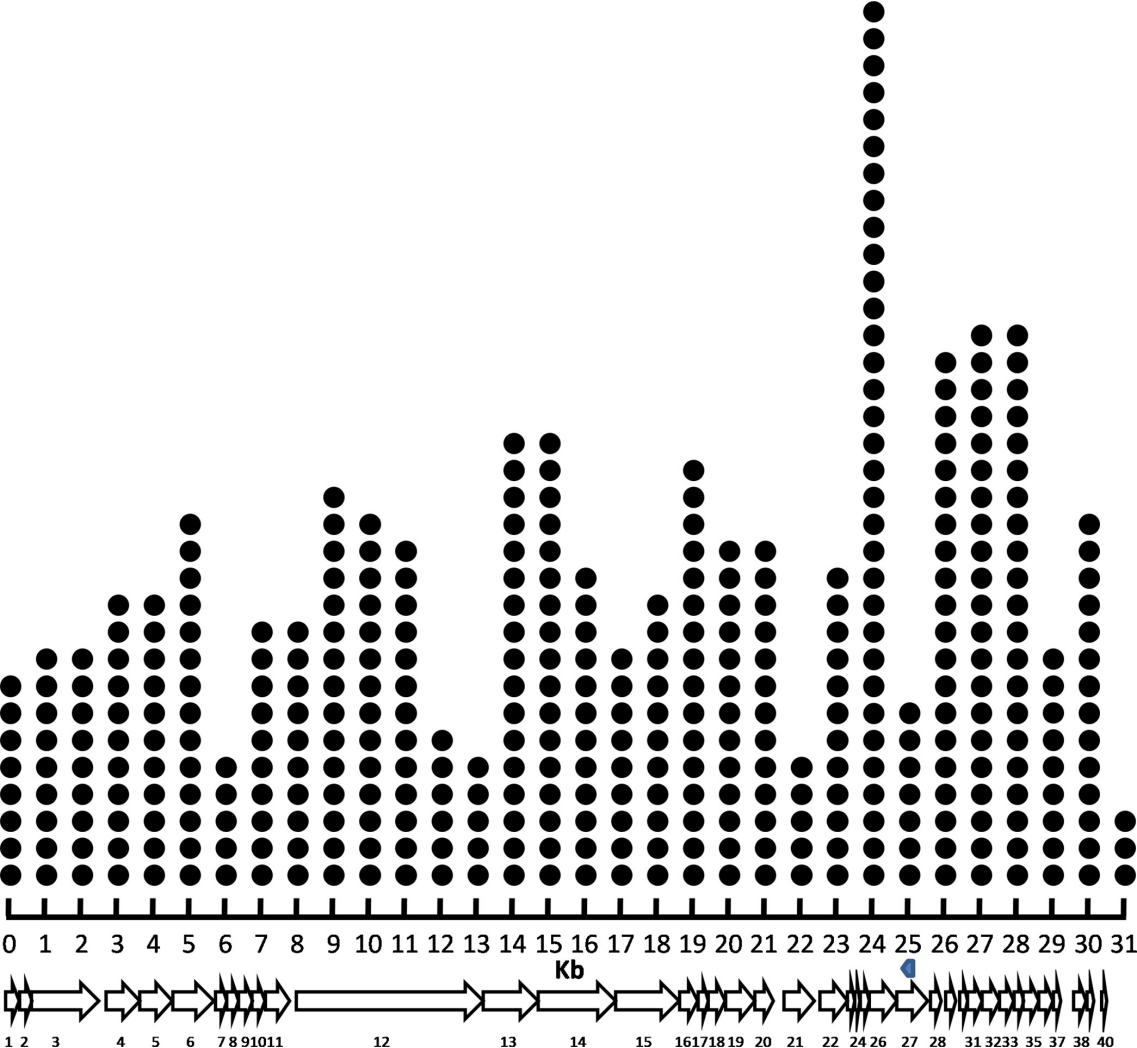
Positions of protospacers on the phage M102AD genome. Each matching spacer was mapped on the 31-kb genome of phage M102AD. For example, at the 1-kb position, each spacer matching a protospacer between 500 and 1,499 bp is indicated by a single dot. At the bottom, the 40 ORFs of M102AD are highlighted as white arrows. The partially matching target of WT spacer 3 is highlighted by a blue arrow.

The distribution of the matching 393 spacers was also mapped on the phage M102AD genome (Fig. 7). When considering the region of the phage M102AD genome partially matching spacer 3, 24% (93/393) of the protospacers were found within 2.5 kb of this region.

### Impact of the multiplicity of phage infection on spacer acquisition.

In a previous study, spacer acquisition was found in 20% of the screened BIMs of S. mutans P42S (14). Here, we investigated in greater detail whether the multiplicity of infection (MOI) had an impact on spacer acquisition. In this study, spacer acquisition was found in 25% of all the BIMs tested. However, more BIMs acquired spacers (37%) when the MOI was between 1 and 10 (Table 2). Multiple-spacer acquisition and ectopic spacer acquisition occurred at all MOIs, with no particular preference (data not shown).

**TABLE 2:** Spacer acquisition in S. mutans P42S after infection with phage M102AD at various MOIs

MOI	No. of BIMs screened	No. (%) of BIMs that have acquired a spacer	No. of BIMs that have ectopically acquired a spacer
<1	189	24 (13)	6
1–10	266	97 (37)	27
>10	225	46 (20)	14

### Phage adsorption assays.

We previously observed that spacer acquisition was often accompanied by reduced phage adsorption in S. mutans (14). We determined the phage adsorption rates for 132 BIMs that were selected at different MOIs (see Table S1 in the supplemental material). When an MOI of >1 was used in phage challenge assays, 62% (72/117) of the BIMs that had acquired at least one new spacer also displayed reduced phage adsorption (Table 3). On the other hand, when lower MOIs of <1 were used, reduced phage adsorption was rarely observed in BIMs that had acquired a new spacer. Plaque assays were performed with 27 BIMs, which still allowed phage adsorption, and they were fully resistant to phage M102AD.

**TABLE 3:** Percentages of adsorption of phage M102AD to various BIMs obtained at different MOIs

MOI^*a*^	No. of BIMs tested	No. (%) of BIMs with <80% phage adsorption
<1	16	2 (12.5)
1–10	72	46 (63.9)
>10	45	26 (57.8)

a MOIs at which the BIMs were obtained.

10.1128/mSphere.00185-21.1 TABLE S1 Adsorption percentages in all strains tested in this study. Download Table S1, DOCX file, 0.02 MB. Copyright © 2021 Mosterd and Moineau. 2021 Mosterd and Moineau. https://creativecommons.org/licenses/by/4.0/ This content is distributed under the terms of the Creative Commons Attribution 4.0 International license.

## DISCUSSION

The adaptation and interference activities of the type II-A CRISPR-Cas system of S. mutans P42S were previously demonstrated (14). Spacer sizes and PAM preferences of this CRISPR-Cas system were confirmed in this study. One key observation here was that the PAM 5′-TAAAA-3′ (17%) was apparently more often used than 5′-TAAAT-3′ (13%). However, it should also be noted that the 5′-TAAAA-3′ motif is found 212 times in the genome of phage M102AD, compared to 81 times for 5′-TAAAT-3′. Nonetheless, these two PAMs were most commonly found to flank protospacers (Table 1).

Of the spacers acquired by 293 BIMs, 88% were identical to a region of the phage M102AD genome, with most of the remaining spacers having one to three mismatches. These mismatches were usually found in the first base pair of the spacer, furthest away from the PAM, and they still had a 30-bp stretch that was identical to phage M102AD, likely indicating that they are still providing interference activity (30). Non-perfectly matching spacers were often found in BIMs that have acquired multiple spacers perfectly matching the genome of phage M102AD. The few other BIMs that acquired only a nonmatching spacer were likely resistant to phages due to other antiviral resistance mechanisms.

An interesting observation was that all 31-bp spacers with a mismatch with the genome of phage M102AD had a C at the first nucleotide, and all 32-bp spacers with a mismatch at the first 2 nucleotides had AC at these positions. The AC motif is also found in the flanking repeat sequences, and therefore, these additional bases may be the result of an error during the replication of the repeat sequence during spacer integration.

The interference activity of some of the non-perfectly matching spacers was tested using a plasmid transformation assay. Spacer 157 had a mismatch at position 12, leaving an 18-bp stretch of sequence at the 3′ end of the spacer that was identical to the targeted double-stranded DNA (dsDNA). Spacer 314 had mismatches at positions 1, 9, and 13, still resulting in a 17-bp stretch of sequence identity at the 3′ end of the spacer. Both plasmids carrying a mismatched protospacer could not be transformed into the BIMs carrying the phage-derived spacer, indicating that the short stretches of sequence identity are sufficient to provide interference. It has been previously shown that within a spacer at the 3′ end, there is a short sequence called the seed sequence, which is essential for spacer target specificity. This phenomenon was observed in type I (31) and type II (32) systems. The size of this seed sequence in type II systems has been defined as being 12 to 13 bp (30, 33–36).

Still, 12% of the acquired spacers were not identical to phage M102AD, perhaps suggesting an error rate in the acquisition machinery of the CRISPR-Cas system of S. mutans P42S. We cannot exclude that these spacers originated from phage mutants that may have been present at a low frequency in the lysate of phage M102AD (37). Two spacers had mismatches compared to the phage M102AD genome but were identical to the closely related phage M102, which was not used in this study and is unable to infect S. mutans P42S (38). Two spacers that did not match the phage M102AD genome were identical to the genome of S. mutans P42S. The sequences on the bacterial genome were not flanked by an appropriate PAM, explaining the viability of these BIMs but not why they were acquired in the first place.

The acquisition of multiple spacers in the type II-A-associated CRISPR array has been observed previously in S. mutans P42S during infection by phage M102AD (14). However, we even isolated a BIM that acquired 10 new spacers, tripling the size of the native array from 5 to 15 spacers. One possible explanation for the acquisition of multiple spacers is that when phage exposure occurs on solid media, the ongoing interaction between BIMs and CRISPR-escaping phages likely results in several rounds of infection and, thus, the acquisition of multiple spacers (24). It has been shown by others that the acquisition of multiple spacers leads to increased phage resistance (4, 20).

We also noticed spacer deletions in BIMs of S. mutans P42S. Spacer deletion is likely why the CRISPR locus of this strain remains relatively small, even though it can actively acquire multiple spacers. Others have shown that an increase in the number of spacers has no major impact on the overall growth fitness of the BIMs, at least up to four new spacers (39), possibly ruling out the hypothesis that the deletion events occurred to increase overall fitness. S. mutans is also a naturally competent species, and the CRISPR-Cas system may interfere with the uptake of foreign DNA. It has been proposed that the deletion of spacers is a mechanism by which bacteria circumvent this downside of CRISPR-Cas (40–42). It has been reported in S. thermophilus that most deletion events take place closer to the 3′ end of the CRISPR locus, perhaps because these ancient spacers protect against foreign nucleic acids that no longer pose a threat (43). Yet in Lactobacillus gasseri, deletion events appear to be more common at the 5′ end of the CRISPR locus (41). In two S. mutans P42S BIMs, the four spacers at the 5′ end of the CRISPR locus were deleted, leaving only the last spacer at the 3′ end. Deletion events have been proposed to be the result of recombination between repeat sequences (40, 44). The last repeat of the CRISPR locus carries a mutation in its last nucleotide, perhaps reducing the possibility of recombination between the final repeat and the previous repeats within the locus.

Even though priming has been primarily associated with type I CRISPR-Cas systems, it has also recently been observed in some type II-A systems (22, 23) albeit at a lower frequency. We investigated if priming may have occurred in the type II-A system of S. mutans. Almost one-third of the acquired spacers were found within 2.5 kb of the first acquired (proto)spacer, and over 10% were within 500 bp. Considering the size of the phage M102AD genome (30,664 bp), our data suggest a preference of acquiring new spacers from protospacers that are within the vicinity of the first acquired (proto)spacer. This trend was even more pronounced (35% within 2.5 kb) when considering only the spacers that were acquired from the opposing strand. In addition, when comparing the distances of this first acquired spacer in relation to an M102AD genomic fragment partially matching native spacer 3 of S. mutans P42S, more than one-half (55%) of the first acquired spacers were found within 2.5 kb from this genomic fragment. Mapping all 393 acquired protospacers showed that more protospacers were found around this specific fragment, as 24% were found within 2.5 kb from this genomic region. Otherwise, protospacers were evenly divided over the phage genome. Taken together, it is likely that priming plays a role in multiple-spacer acquisition in S. mutans P42S.

In type I CRISPR-Cas systems, priming requires the activity of Cas3 to generate substrates for subsequent spacer acquisition events (17, 18, 21). In the type II-A system, Cas9 nuclease activity may also generate substrates leading to an increase in spacer acquisition (23). Cas9 has been reported to remain bound to its cleavage products (36), from where it could perhaps guide the acquisition complex to a new acquisition target (23).

Primed spacer acquisition was originally thought to occur only from the same strand as the original spacer in type I systems (17, 18, 21). However, priming has been observed without strand bias (16, 19). In type II-A systems, strand bias was not detected in previous studies (22, 23) and in this study. Priming typically occurs in type I systems when the original spacer has mismatches with the targeting protospacer or a suboptimal PAM (19, 21), while priming appears to occur with fully functional spacers in type II-A systems (23). In S. mutans P42S, the first acquired spacer was typically identical to a region in the phage genome and flanked by a functional PAM. Yet the native spacer 3 has partial identity with the phage M102AD genome, and it is flanked by the functional PAM 5′-TAAAC-3′. Despite the lack of interference activity, this spacer may play a role in priming.

In addition to CRISPR-Cas systems, reduced phage adsorption also plays a role in the phage resistance of S. mutans (14). When we used a lower MOI, the BIMs acquired spacers but mostly did not concomitantly impair phage adsorption. However, at higher MOIs, a secondary phage defense mechanism emerged in the BIMs, likely affecting the unknown phage receptors on S. mutans P42S. Presumably, this additional resistance is to cope with the higher number of phages. Further studies are needed to determine if other host factors are also at play (1, 45).

## MATERIALS AND METHODS

### Bacterial strain, phage, and culture conditions.

The wild-type strain S. mutans P42S (38, 46) and the virulent phage M102AD (*Siphoviridae*) were obtained from the Félix d’Hérelle Reference Center for Bacterial Viruses (www.phage.ulaval.ca). The conditions to grow the bacterial strains and amplify the phage were described previously (14).

### BIM assays.

A culture of S. mutans P42S grown overnight was transferred (1%) to fresh brain heart infusion (BHI) medium and grown until an optical density at 600 nm (OD_600_) of 0.4 was reached. The bacterial culture was then mixed in BHI top agar with the appropriate PFU per milliliter of phage M102AD to obtain the desired MOI. The mixture was poured on solid medium, and plates were incubated at 37°C for 72 h. Surviving cells were analyzed for spacer acquisition through the amplification of the CRISPR locus using the primers CR-F (5′-AATGTCGTGACGAAAATTGG-3′) and CR-R (5′-GAAGTCATCGGAACGGTCAT-3′). PCR products were sequenced using an ABI 3730xl analyzer at the Centre de Génomique du CHU de Québec-Université Laval.

### Plasmid interference assays.

Plasmid interference assays were performed as described previously (14). Plasmid constructs were prepared using the vector pNZ123 (47), which contains a chloramphenicol resistance gene and is transformable in S. mutans. The 24 bp between the XhoI and EcoRI restriction sites were removed, and the linearized plasmid was purified from an agarose gel using the QIAquick gel purification kit as described by the manufacturer. DNA inserts were ligated between these restriction sites. These DNA inserts consisted of sequences from the phage M102AD genome that were targeted by various spacers.

The first insert was the target of spacer 157. This 30-bp spacer matches a region of the phage M102AD genome (positions 19006 to 18977) in 29 out of 30 bp and had a mismatch at position 12. The protospacer in the M102AD genome is flanked by the PAM 5′-TAAAG-3′. This 30-bp target and the flanking PAM were cloned between the XhoI and EcoRI restriction sites of pNZ123 to generate pNZsp157. A second insert was the target of spacer 314, a 30-bp-long spacer with mismatches at positions 1, 9, and 13 compared to the genome of M102AD. The spacer targets a 30-bp stretch on the M102AD genome (positions 21055 to 21028) that is flanked by 5′-TAAAA-3′. This targeted sequence and PAM were cloned into pNZ123 to generate pNZsp314. Clones were confirmed by sequencing, and the sequences of the inserts of the constructs are shown in Table 4.

**TABLE 4:** Sequences of the protospacers and PAMs cloned into pNZ123^*a*^

Construct	Insert sequence (5′–3′)	Targeting spacer sequence (5′–3′)
pNZsp157	TCCACTAATTTCGTCATCACTAAAATCAAC*TAAAG*	TCCACTAATTTTGTCATCACTAAAATCAAC
pNZsp314	GATACAAACAATAAACTAGCTGACAAACC*TAAAA*	TGATACAATCAACAAACTAGCTGACAAACC

a The PAM sequences are in italics. Mismatches between the protospacer and the spacer sequences are underlined.

Constructs were transformed into S. mutans P42S using natural competence (48). A culture of S. mutans P42S grown overnight in sterile-filtered tryptic soy broth supplemented with 0.5% yeast extract and 0.5% K_2_HPO_4_ (TSYE) was transferred to fresh medium and grown at 37°C until the OD_600_ reached 0.1. Next, aliquots of 500 μl were collected, and 10 μg of plasmid DNA was added to the aliquots. The cultures were incubated at 37°C for 4 h and spun down, and the cell pellets were resuspended in 100 μl of TSYE medium. Samples were plated onto TSYE agar plates supplemented with 5 μg/ml chloramphenicol. Plates were incubated at 37°C for 120 to 168 h.

### Phage adsorption assay.

A culture of S. mutans P42S grown overnight was transferred (2%) to fresh BHI medium and grown until an OD_600_ of 0.7 was reached. Phage M102AD (10^3^ PFU) was added to 900 μl of this culture and allowed to adsorb for 15 min at 37°C. Cultures were then centrifuged for 1 min at 13,200 rpm, and the titer of the supernatant was determined to determine the fraction of the phages that did not adsorb to the host cells. A BIM was considered to have a reduced phage adsorption phenotype if <80% of the added phage particles had adsorbed to the BIM.
