# Supplementary material for: Primed CRISPR-Cas Adaptation and Impaired Phage Adsorption in Streptococcus mutans
Source: mSphere. 2021 May 19;6(3):e00185-21. doi: 10.1128/mSphere.00185-21 (PMC8265633; doi:10.1128/mSphere.00185-21)
Supplement: TABLE S1 [file msphere.00185-21-st001.docx]

# Supplementary Data

Table S1: Adsorption of phage M102AD on various strains used in this study. BIM5 was obtained in a previous study (14). In the first and third columns, the new BIMs are identified by the MOI at which they were obtained, followed by the colony number. The second and fourth columns indicates the adsorption of phage M102AD to the strain, expressed in percentages and accompanied by the standard deviation.

| **Strain** | **Phage adsorption (%)** |  | **Strain** | **Phage adsorption (%)** |
| --- | --- | --- | --- | --- |
| WT | 97.4 ± 2.2 |  | 2.2 – 42A | 59.5 ± 0.5 |
| BIM5 | 75.1 ± 17 |  | 2.2 – 43CA | 93.0± 1.0 |
| 0.07 – 27 | 98.8 ± 0.4 |  | 2.2 – 44C | 57.0± 0.0 |
| 0.07 – 33 | 98.2 ± 1.3 |  | 2.2 – 45B | 83.0± 0.0 |
| 0.07 – 35 | 97.5 ± 0.5 |  | 2.2 – 45C | 69.5 ± 2.5 |
| 0.07 – 37 | 98.0 ± 1.9 |  | 2.2 – 46 | 98.0± 0.0 |
| 0.07 – 45 | 96.8 ± 2.5 |  | 2.2 – 48 | 74.0± 2.0 |
| 0.07 – 47 | 94.0 ± 0.0 |  | 4.6 – 8 | 15.0± 10.0 |
| 0.07 – 51 | 96.0 ± 0.0 |  | 4.6 – 9B | 98.5 ± 0.5 |
| 0.07 – 59 | 97.5 ± 0.5 |  | 4.6 – 9C | 65.0± 2.0 |
| 0.14 – 22 | 98.5 ± 0.5 |  | 4.6 – 10 | 97.5 ± 0.5 |
| 0.14 – 35 | 98.0± 0.0 |  | 4.6 – 13 | 76.0± 6.0 |
| 0.68 – 26 | 96.0 ± 0.0 |  | 4.6 – 14 | 26.0± 13.0 |
| 0.68 – 49 | 92.0± 4.0 |  | 4.6 – 15 | 20.5 ± 9.5 |
| 0.68 – 50 | 97.0± 1.0 |  | 4.6 – 16 | 98.5 ± 0.5 |
| 0.68 – 51 | 94.5 ± 1.5 |  | 4.6 – 18 | 48.0± 11.0 |
| 0.68 – 58 | 44.0± 11.0 |  | 4.6 – 23 | 49.5 ± 3.5 |
| 0.68 – 59 | 44.5 ± 1.5 |  | 4.6 – 25 | 97.0± 0.0 |
| 1.4 – 9 | 26.0 ± 6.0 |  | 4.6 – 27 | 42.0± 3.0 |
| 1.4 – 15 | 98.0 ± 1.0 |  | 4.6 – 29C | 98.5 ± 0.5 |
| 1.4 – 16 | 0.0± 0.0 |  | 4.6 – 34 | 84.0± 2.0 |
| 1.4 – 17 | 97.5 ± 0.5 |  | 4.6 – 35 | 34.0± 3.0 |
| 1.4 – 28 | 98.5 ± 0.5 |  | 4.6 – 36 | 40.0± 3.0 |
| 2.2 – 6AB | 12.5 ± 6.5 |  | 4.6 – 37C | 14.0± 12.0 |
| 2.2 – 6AC | 23.5 ± 14.5 |  | 4.6 – 40 | 26.0± 8.0 |
| 2.2 – 6B | 98.5 ± 0.5 |  | 4.6 – 41 | 97.5 ± 0.5 |
| 2.2 – 7 | 99.0± 0.0 |  | 6.8 – 8 | 98.0 ± 1.0 |
| 2.2 – 11 | 67.5 ± 4.5 |  | 6.8 – 14 | 23.0 ± 1.0 |
| 2.2 – 12 | 70.5 ± 1.5 |  | 6.8 – 16 | 56.0 ± 11.0 |
| 2.2 – 17 | 19.0± 11.0 |  | 6.8 – 18 | 98.5 ± 0.5 |
| 2.2 – 20A | 65.0± 3.0 |  | 6.8 – 26 | 27.5 ± 27.5 |
| 2.2 – 20C | 62.0± 5.0 |  | 6.8 – 28 | 58.5 ± 0.5 |
| 2.2 – 29B | 54.0± 6.0 |  | 6.8 – 33 | 97.5 ± 0.5 |
| 2.2 – 29C | 46.0± 2.0 |  | 6.8 – 38 | 95.5 ± 0.5 |
| 2.2 – 40A | 24.5 ± 23.5 |  | 9.2 – 1A | 27.0± 3.0 |
| 2.2 – 40C | 47.5 ± 1.5 |  | 9.2 – 3 | 96.5 ± 2.5 |
| **Strain** | **Phage adsorption (%)** |  | **Strain** | **Phage adsorption (%)** |
| 9.2 – 5 | 54.0± 2.0 |  | 26.7 – 67A | 12.5 ± 12.5 |
| 9.2 – 6 | 45.0± 6.0 |  | 26.7 – 67B | 4.5 ± 4.5 |
| 9.2 – 8 | 68.0± 11.0 |  | 26.7 – 70B | 26 ± 27.2 |
| 9.2 – 8A | 98.5 ± 0.5 |  | 26.7 – 73C | 95.5 ± 2.5 |
| 9.2 – 8C | 98.0± 1.0 |  | 26.7 – 78A | 0.0± 0.0 |
| 9.2 – 10 | 37.5 ± 17.5 |  | 26.7 – 82A | 93.0± 0.0 |
| 9.2 – 13 | 38.5 ± 16.5 |  | 26.7 – 85A | 93.0± 0.0 |
| 9.2 – 14 | 43.5 ± 6.5 |  | 26.7 – 85B | 91.0± 2.0 |
| 9.2 – 15 | 99.0± 0.0 |  | 26.7 – 85C | 93.5 ± 2.5 |
| 9.2 – 16 | 99.0± 1.0 |  | 27.5 – 3A | 25.0± 6.0 |
| 9.2 – 20 | 19.0± 3.0 |  | 27.5 – 19 | 42.0± 4.0 |
| 9.2 – 21 | 99.0± 0.0 |  | 27.5 – 25A | 63.0± 0.0 |
| 9.2 – 22 | 94.5 ± 2.5 |  | 53.3 – 4B | 96.5 ± 1.5 |
| 9.2 – 27 | 98.5 ± 0.5 |  | 53.3 – 4C | 97.5 ± 1.5 |
| 9.2 – 31 | 15.5 ± 15.5 |  | 53.3 – 28A | 27.5 ± 9.4 |
| 9.2 – 43 | 25.5 ± 9.5 |  | 53.3 – 54A | 96.5 ± 3.5 |
| 9.2 – 44 | 99.0± 0.0 |  | 53.3 – 57A | 92.5 ± 1.5 |
| 9.2 – 46 | 42.5 ± 9.5 |  | 53.3 – 57B | 97.0± 0.0 |
| 18.3 – 4 | 82.0± 2.0 |  | 53.3 – 65A | 92.5 ± 4.5 |
| 18.3 – 8 | 58.0± 9.0 |  | 53.3 – 65C | 96.5 ± 0.5 |
| 18.3 – 12 | 92.5 ± 1.5 |  | 53.3 – 76B | 96.8 ± 2.5 |
| 18.3 – 14 | 97.5 ± 1.5 |  | 53.3 – 79F | 92.5 ± 4.5 |
| 18.3 – 15 | 33.5 ± 1.5 |  | 53.3 – 82B | 29.8 ± 18.8 |
| 26.7 – 18A | 61.0± 2.0 |  | 53.3 – 83A | 24.0± 8.0 |
| 26.7 – 25B | 97.5 ± 0.5 |  | 53.3 – 83B | 26.0± 5.0 |
| 26.7 – 27A | 0.0± 0.0 |  | 53.3 – 83C | 5.0± 1.0 |
| 26.7 – 27C | 15.3 ± 20.3 |  | 53.3 – 85B | 14.0± 5.0 |
| 26.7 – 31A | 95.5 ± 1.5 |  | 53.3 – 87A | 1.0 ± 0.0 |
| 26.7 – 50B | 97.0± 1.0 |  | 53.3 – 90A | 27.0± 8.0 |
| 26.7 – 52A | 29.0± 8.0 |  | 53.3 – 90B | 25.5 ± 9.5 |
| 26.7 – 52B | 27.0± 7.0 |  | 53.3 – 90C | 24.5 ± 2.5 |
| 26.7 – 56A | 1.5 ± 0.5 |  |  |  |
